# Effect of Mechanical Activation of Al-Ti-B Powder Mixture on Phase Composition and Structure of Al-TiB_2_ Composite Materials Obtained by Self-Propagating High-Temperature Synthesis (SHS)

**DOI:** 10.3390/ma15072668

**Published:** 2022-04-05

**Authors:** Alexey Matveev, Vladimir Promakhov, Pavel Nikitin, Artem Babaev, Alexander Vorozhtsov

**Affiliations:** Center for Additive Technologies, National Research Tomsk State University, Lenin Avenue, 36, 634050 Tomsk, Russia; vvpromakhov@mail.ru (V.P.); upavelru@yandex.ru (P.N.); temkams@mail.ru (A.B.); abv1953@mail.ru (A.V.)

**Keywords:** mechanical activation, self-propagating high-temperature synthesis, non-steady combustion, phase composition, titanium diboride, aluminum

## Abstract

In this study, we successfully obtained Al-TiB_2_ composite materials using self-propagating high-temperature synthesis and preliminary mechanical activation of the initial Al-(Ti + 2B) powder mixture with a high aluminum content (70 wt.%). We investigated the possibility of controlling the structure of synthesis products, in particular, the size and shape of ceramic particles. We examined the effects of the mechanical activation of the initial powder mixture on the structure and particle size of titanium diboride in the synthesis products. We proposed a mechanism of structure formation in the synthesis products obtained by SHS using the method of preliminary mechanical activation of the initial mixture. We found that mechanical activation for 60-180 s led to the formation of isolated TiB_2_ particles of prolate and irregular shape. The average particle size of TiB_2_ in the synthesis products was 0.77 (after 60 s of mechanical activation) and 1.5 µm (after 180 s of mechanical activation), respectively. An increase in the duration of mechanical activation to 900 s led to the formation of an island (skeletal) structure, in which there were interconnected aggregates and isolated particles of titanium diboride. The average size of these particles was 4.3 µm.

## 1. Introduction

Self-propagating high-temperature synthesis (SHS) is a technology based on localized exothermic reactions (with heat release) that spontaneously propagate throughout the sample. A synthesis reaction propagates throughout the sample in the form of a solid-flame wave of layer-by-layer, self-oscillatory, or spin combustion [1]. The SHS method was developed in 1967 by A.G. Merzhanov, I.P. Borovinskaya, V.M. Shkiro, and others, during an experimental study of the combustion model of condensed systems [2]. Self-propagating high-temperature synthesis was used to obtain classes of compounds such as carbides, borides, silicates, nitrides, intermetallic compounds, etc. [3]. Today, scientists are focusing their attention on the possibility of using the SHS method to obtain composite metal-matrix materials [4,5,6]. These composites consist of ceramic particles that are uniformly distributed throughout a metal matrix [7]. Due to the presence of ceramic inclusions in the metal-matrix composites, their properties (hardness, strength, operating temperatures, and oxidation resistance) are improved relative to traditional metallic materials [8]. In a previous study [9], we used the SHS method to obtain composite structured particles from (Ni-Ti)-TiB_2_ powders. It was found that the particle structure consisted of an Ni-Ti intermetallic matrix, in which TiB_2_ particles were uniformly distributed. The particle size varied from 0.1 to 4 µm, and their average size was 0.57 µm. In another study [10], we synthesized composite structured particle powders of CrNi-TiN using the SHS method. The particle structure consisted of titanium nitride (TiN) ceramic inclusions, which were uniformly distributed throughout the CrNi intermetallic matrix. The particle size varied from 0.02 to 5 µm, and the average particle size was 1.1 µm. CrNi-TiN powders were successfully used as feedstock for the production of composite materials by direct laser growth. The hardness of such materials is higher than the hardness of analogues obtained from Inconel powder [10]. In [9,10], the authors found that ceramic particles in the metal matrix composites of CrNi-TiN and (Ni-Ti)-TiB_2_ were formed during SHS (in situ) due to exothermic reactions of the initial components of the NiB-Ti and CrN-TiNi powder mixtures.

Controlling exothermic reactions by setting the initial synthesis parameters (synthesis conditions, composition of the initial mixture, etc.) makes it possible to also control the phase composition, structure (including the particle size of the ceramic phase), and, consequently, properties of the obtained composite materials [11,12,13]. For example, in [14], TiB_2_-TiC-Co composite materials were obtained using the SHS method from a Ti-B_4_C-Co mixture with a Co content of 10-60 wt.%. It was found that an increase in the cobalt content led to a decrease in the combustion temperature of the samples. The synthesis products consisted of irregularly and near-spherical shaped titanium diboride particles, which were distributed throughout the cobalt matrix. The authors also showed that an increase in cobalt content in the initial mixture from 10 to 50 wt.% led to a decrease in the size of ceramic particles, from 3–12 µm to 1–4 µm.

In [15], the authors demonstrated the possibility of using thermochemically coupled self-propagating high-temperature synthesis to obtain a composite ceramic material AlMgB_14_-TiB_2_ from a (Al_12_Mg_17_-B)-(Ti + 2B) powder mixture. They showed that the weakly exothermic AlMgB_14_ compound is formed from the Al_12_Mg_17_-B powder mixture (acceptor) under the heat released during the exothermic reaction of the (Ti + 2B) mixture (donor). The obtained composite material consisted of titanium diboride particles and aggregates, which formed a bonded island structure inside the AlMgB_14_ matrix. In this case, as shown in [16], a change in the concentration of titanium and boron components in the initial (Al_12_Mg_17_-B)-(Ti + 2B) mixture led to a change in the phase composition and structure of the SHS composite.

Composites consisting of an aluminum matrix and ceramic particles are some of the most promising materials. They have a low density and the ceramic particles in these composites have increased physical and mechanical properties relative to aluminum alloys without particles [17,18,19]. Such composites can be used in the production of engine parts (cylinder liners, pistons, etc.), and other car parts as well, reducing the total mass of the parts while maintaining the required properties (hardness, strength, wear resistance, etc.) [20]. In [21], the authors successfully applied the SHS method to obtain an (Al-Ti)-TiB_2_ metal matrix composite from an Al-Ti-B powder mixture. They found that an increase in the Al mass content in the Al-Ti-B mixture from 10 to 50 wt.% led to a decrease in the content of TiB_2_ ceramic particles in the obtained (Al-Ti)-TiB_2_ composites, from 80 to 50 wt.%, and reduced their size from 4 to 0.8 µm. Thus, it has been shown that changing the ratio of components in the initial powder mixture makes it possible to control the size of ceramic particles in the (Al-Ti)-TiB_2_ SHS composites. It should also be noted that a decrease in the size of ceramic particles makes it possible to achieve an optimal combination of physical and mechanical properties in aluminum-based composites [22,23]. However, a high content of ceramic phase (50 wt.% or more) in the SHS composites leads to the occurrence of large internal stresses, which contribute to the formation of cracks in these materials and their brittle fracture [24]. In this case, an increase in the inert component in the initial mixture makes it difficult or impossible for spontaneous exothermic reactions to occur during SHS, as the heat that is released from exothermic reactions is used heating and melting the inert component to form a matrix in the composites. Therefore, an increase in the content of inert component in the initial mixture leads to an increase in absorbed heat. This hinders the synthesis processes and can lead to flame front damping or an inability for the synthesis processes to initiate. To avoid this, preheating of the initial powder mixture is performed to increase its initial temperature and supply an additional heat source [25].

In our previous study [26], we successfully used mechanical activation of the initial Al-Ti-B powder mixture with an aluminum concentration of 60 wt.% to obtain metal matrix composite materials using SHS. SHS synthesis of this mixture was impossible without mechanical activation. We found that mechanical activation with a duration of 60–900 s led to the grinding of the initial components in the Al-Ti-B mixture and the formation of complex particles. Each complex particle contained fine particles of aluminum, titanium, and boron. This made it possible to increase the reaction surface of the entire system and to carry out the synthesis reaction in the system. We found that an increase in the mechanical activation duration from 60 to 900 s led to an increase in the average particle size of TiB_2_, from 0.26 to 0.4 µm. The obtained results demonstrate that the use of mechanical activation makes it possible to initiate synthesis in materials with a high content of inert component (for example, aluminum powder) and to control the synthesis processes and the size of ceramic particles in the obtained SHS materials. The question then arises of what occurs when using mechanical activation on an Al-Ti-B system with a higher Al content of 70–80 wt.% to obtain SHS composites. In this case, it is possible to reduce the content of the TiB_2_ ceramic phase and, consequently, to reduce internal stresses in SHS composites obtained from this powder mixture. Thus, the purpose of this study is to examine the effects of the mechanical activation duration of the initial Al-Ti-B powder mixture with an aluminum concentration of 70 and 80 wt.% on the synthesis processes, phase composition, and structure of the obtained SHS composites.

## 2. Materials and Methods

**Figure 1:**
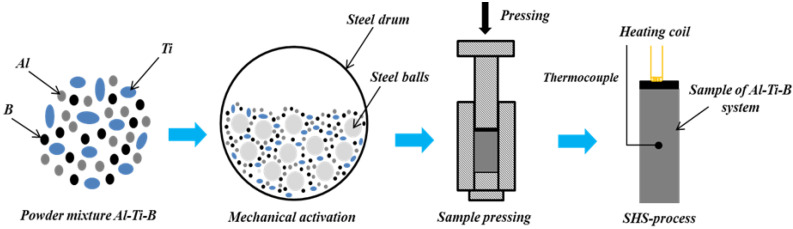
Representation of the Al-Ti-B powder mixture sample preparation process.

Figure 1 shows the Al-Ti-B powder mixture sample preparation process. Powders of titanium (OJSC Polema, Tula, Russia) (average particle size of 140 μm) and boron (OJCS Aviabor, Dzershinsk, Nizhny Novgorod region, Russia) (average particle size of 0.6 μm) were mixed in a stoichiometric ratio to obtain titanium diboride (Ti + 2B → TiB_2_): 69 wt.% Ti + 31 wt.% B [11]. Aluminum powder (Sual-PM Ltd, Shelkhov, Irkutsk region, Russia) (average particle size of 100 μm) was added to the obtained mixture at a ratio of 70 wt.% of the total mixture.

The powders were mixed in ethanol, and then the mixture was dried in a vacuum furnace at 60 °C for 1 h and at 200 °C for 3 h. The dried powder mixture was mechanically activated in a planetary mill (Chemical Engineering Plant LTD, Novosibirsk, Russia). Steel balls (Chemical Engineering Plant LTD, Novosibirsk, Russia), with a diameter of 5 mm, were used as grinding bodies. The mass ratio of mixture to balls was 1 to 30. The rotational frequency was 15 Hz. The mechanical activation duration for each Al-Ti-B powder mixture was 60, 180, and 900 s, respectively.

In the next stage, cylindrical samples with a diameter of 23 mm and a mass of 25 g were cold-pressed from the obtained mixtures. The pressure for all samples was 215 MPa. The obtained samples were placed in a reactor. The reactor (Chemical Engineering Plant LTD, Novosibirsk, Russia) was evacuated and filled with argon at a pressure of 5 atm. The synthesis reaction was initiated by local heating of the upper surface of the sample with a molybdenum spiral through an igniting layer pressed into the sample. The composition of the igniting layer was 82.8 wt.% Ti + 17 wt.% B. The flame front propagation process was recorded on a video camera. The combustion temperature was measured by introducing tungsten-rhenium thermocouples into the sample and recording the signal in the form of oscillograms on a computer.

The phase composition of the SHS products was studied using a Shimadzu XRD-6000 diffractometer (Shimadzu Corporation, Kyoto, Japan), with filtered CuKα radiation. The crystal lattices of the Powder Diffraction File 4 database were used as references. Calculations of the phase composition, lattice parameters, and CSR sizes were performed by refining the structure using the full-profile analysis method (Rietveld method). The structure of the SHS products was observed by scanning electron microscopy (QUANTA 200 3D, FEI Company, Hillsborough, OR, USA), equipped with energy dispersive spectroscopy (EDS). The size of ceramic particles in the materials was measured with the method of linear intersections using SEM images.

## 3. Results and Discussion

**Figure 2:**
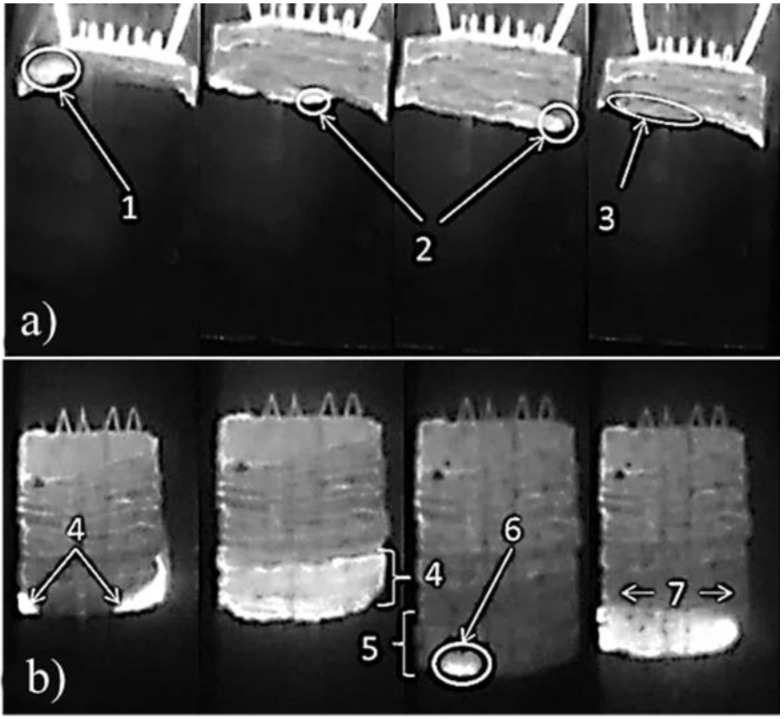
Image of the SHS process in the Al-Ti-B samples: (**a**) after 60 s and (**b**) after 900 s of mechanical activation.

Figure 2a shows a typical image of the SHS process in the Al-Ti-B samples (with the Al content of 70 wt.%) after 60 s of mechanical activation A nucleation site for reaction is formed on the side surface of the sample (region 1), which moves along the spin trajectory (region 2) and makes one revolution along the sample surface. In [27], the authors characterized such a flame front propagation as spin. Under the combustion front, a heated layer zone is formed (region 3), in which a new nucleation site for reaction is formed. The reaction front does not change direction. It should be noted that when increasing the duration of mechanical activation to 180 s, the nature of the propagation of the flame front throughout the sample does not change. One synthesis center is formed, which, as in the previous case, moves along a spin trajectory.

Mechanical activation for 900 s leads to a change in the combustion nature of the system (Figure 2b). Two foci are observed on the lateral surface, which move towards each other (region 4). Under the flame front, a heating zone is formed (region 5), in which a new nucleation site for reaction appears. The nucleation site for reaction spreads over the sample in the form of two waves moving in opposite directions. We found that the thickness of the heating zone and the flame front in this case is greater than in the samples, the mechanical activations of which were 60 and 180 s. Such a combustion regime is characterized as a limiting one, after which attenuation occurs [27].

**Figure 3:**
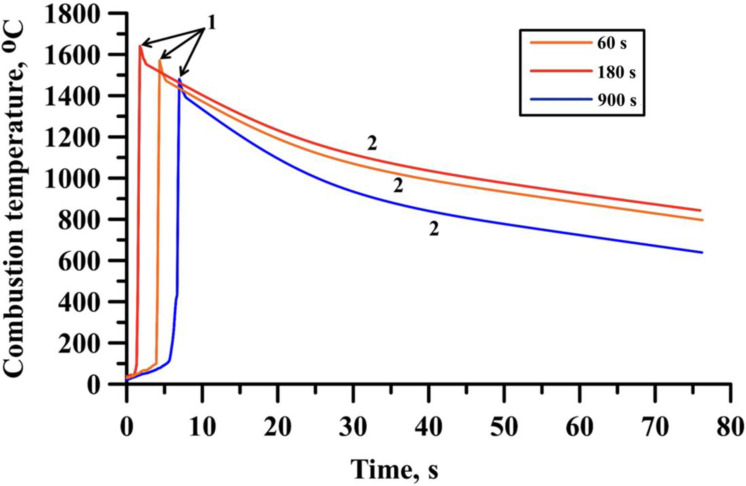
Typical heat patterns of the samples obtained from Al-Ti-B powder mixture after 60, 180, and 900 s of mechanical activation.

Heat patterns of the samples obtained from the Al-Ti-B powder mixture after 60, 180, and 900 s of mechanical activation are shown in Figure 3.

**Figure 4:**
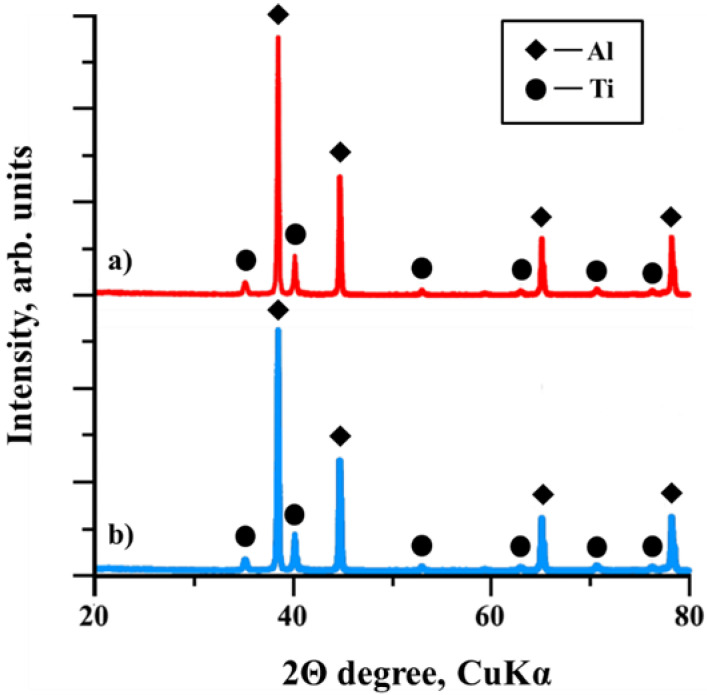
XRD patterns of the Al-Ti-B powder mixture: (**a**) before mechanical activation and (**b**) after 900 s of mechanical activation.

The peaks on the heat patterns (region 1) represent the exothermic reaction of the initial components of the mixtures, which is accompanied by the release of a large amount of heat. The tops of the peaks correspond to the reaction temperature of the components in the Al-Ti-B samples. The temperature then decreases, which represents the cooling of the SHS products (region 2). The heat patterns show that a change in the mechanical activation duration leads to a change in the maximum temperature of the exothermic reaction in the samples. With an increase in the mechanical activation duration from 60 to 180 s, an increase in the synthesis temperature from 1570 °C to 1640 °C is observed. A further increase in the mechanical activation duration to 900 s leads to a decrease in the synthesis temperature to 1480 °C. In our previous study [26], we found that mechanical activation with a duration of 60 s led to the grinding of the initial components in the Al-Ti-B mixture and the formation of complex particles. Each complex particle contained fine particles of aluminum, titanium, and boron. This made it possible to increase the reaction surface of the entire system and to carry out the synthesis reaction. Meanwhile, an increase in the duration of mechanical activation to 180 s led to an even greater grinding of the particles of the initial components, an improvement in the reaction surface and, consequently, an increase in the synthesis temperature. Based on the obtained results and the reported data [26], we supposed that with an increase in the mechanical activation of the Al-Ti-B mixtures containing 70 wt.% aluminum from 60 to 180 s, the interaction surface between the components increases due to the milling of their particles, which directly affects the nature of the flame front movement, as well as the reaction temperature [27]. However, [26] showed that an increase in the mechanical activation to a duration longer than 180 s leads to the formation of large aggregates from the complex particles of the initial Al-Ti-B mixture, which stick together under the influence of van der Waals forces [28], and which in turn leads to a reduced density of the initial samples and reduces the reaction surface. An increase in the duration of mechanical activation of the Al-Ti-B mixture with an aluminum content of 70 wt.% up to a duration of 900 s leads to the formation of such aggregates and a decrease in the density of the samples. In this case, the synthesis reaction is localized in separate foci with a higher contact between the components (that is, the area in which the complex particles in the aggregates are most closely adjacent to each other) [2,27]. The heat from these sources is spent on heating neighboring zones with less tight contact, which leads to an increase in the thickness of the heating zone and duration of heating of the components, a violation of the stability of the flame front propagation (the formation of several foci that move in the opposite direction), and a decrease in the reaction temperature [2,27]. Figure 4 shows the XRD patterns of the initial Al-Ti-B powder mixture (with an Al content of 70 wt.%) before mechanical activation and after 900 s of mechanical activation.

We found that the powder mixture contained Al and Ti phases both before and after mechanical activation. It should be noted that no reflections belonging to the boron phase were found in the XRD pattern, since amorphous boron powder is characterized by the presence of short-range order in the mutual arrangement of its atoms [29], which makes it impossible to classify it as a crystalline phase.

**Figure 5:**
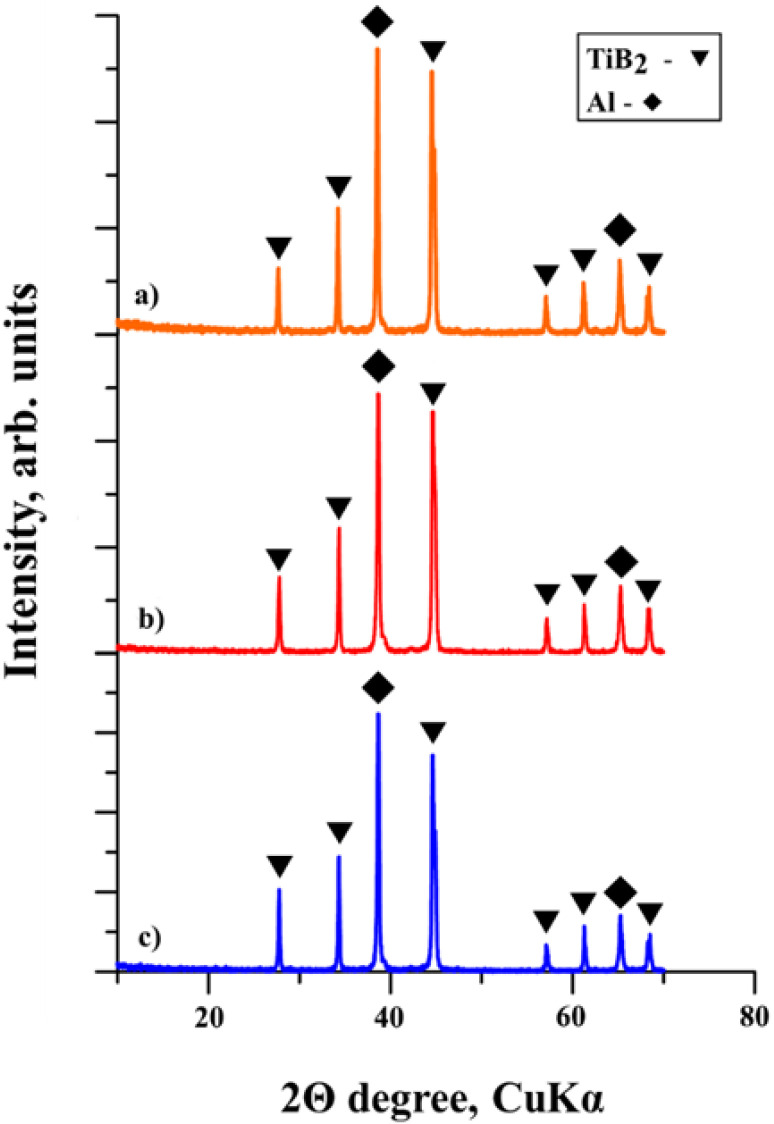
XRD patterns of the SHS products obtained from the Al-Ti-B powder mixture after (**a**) 60 s, (**b**) 180 s, and (**c**) 900 s of mechanical activation.

The XRD patterns of the SHS products obtained from the Al-Ti-B powder mixture after mechanical activation are shown in Figure 5. The XRD results are shown in Table 1.

It was found that all SHS products contain phases of titanium diboride (TiB_2_) and aluminum. The crystal lattice parameters of the presented phases do not qualitatively differ from each other and are close to the lattice parameters of the reference phases of aluminum and titanium diboride [30,31]. The obtained results show that mechanical activation does not affect the formation of new phases in the powder mixture.

**Figure 6:**
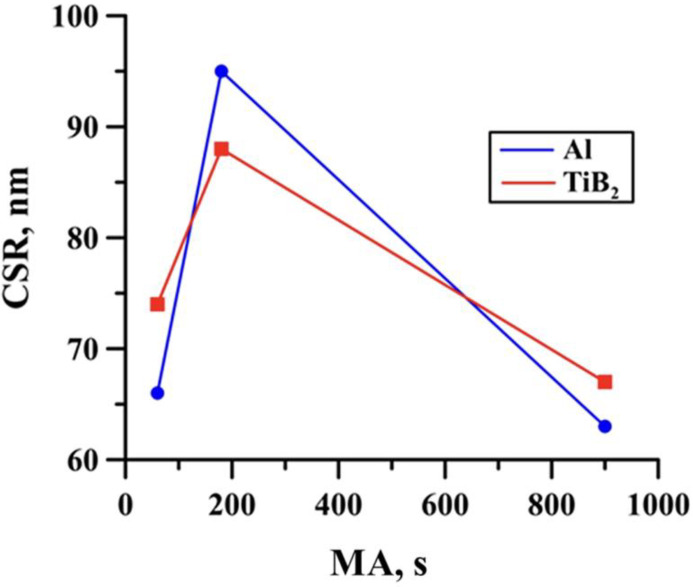
Dependence of the CSR size of the Al and TiB_2_ phases in the SHS products on the mechanical activation duration of the initial Al-Ti-B powder mixture.

The phase contents in the obtained SHS products also do not qualitatively differ from each other and correspond approximately (considering the error of the diffractometer 3–5%) to the mass ratio of the components in the initial mixture. It was also found using X-ray scattering that mechanical activation affects the size of the coherent scattering regions (CSRs) in the SHS products. Figure 6 shows the dependence of the CSR size of the TiB_2_ and Al phases on the mechanical activation duration of the initial mixture. This dependence is related to the reaction temperature of the Al-Ti-B samples. The size of the CSRs characterizes the size of the crystallites in the materials [32]. In addition, [33] suggests that an increase in the synthesis temperature leads to the growth of crystallites in the material. Consequently, mechanical activation of the Al-Ti-B mixture for 180 s leads to the maximum value of the Al and TiB_2_ crystallite size, as well as the CSR size of these phases.

**Figure 7:**
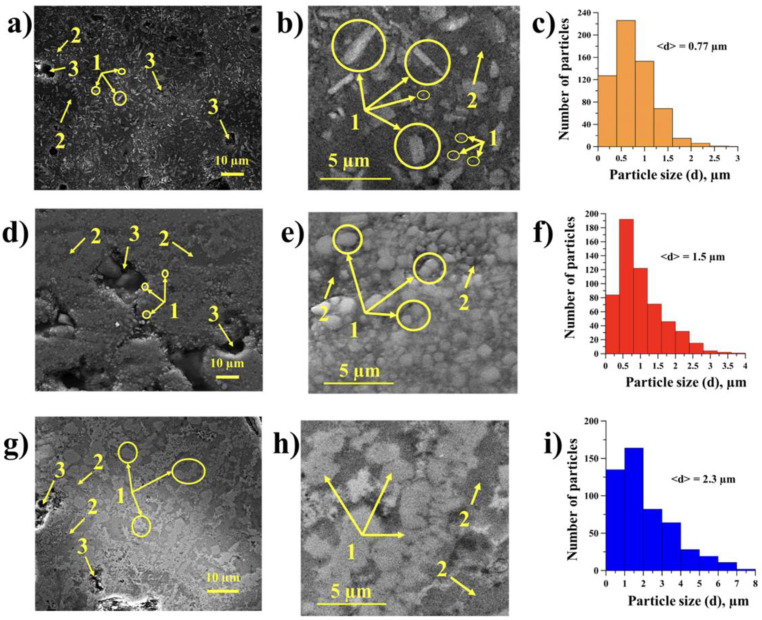
Backscattered SEM images of the SHS products and distribution of TiB_2_ particles in the SHS products obtained from the Al-Ti-B powder mixture after: (**a**–**c**) 60 s, (**d**–**f**) 180 s, and (**g**–**i**) 900 s of mechanical activation. Region 1: TiB_2_ particles; region 2: aluminum matrix; region 3: pores.

**Figure 8:**
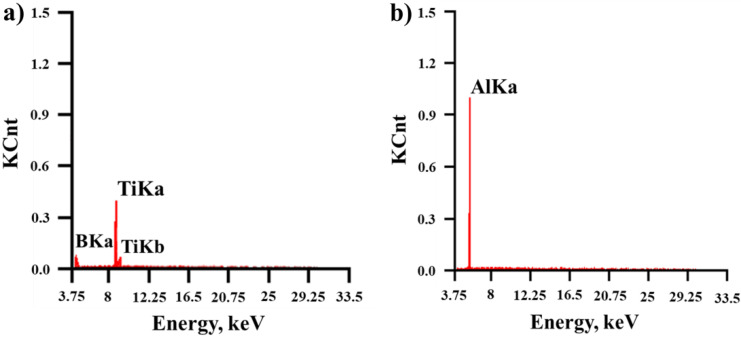
Qualitative elemental analysis of the structural regions of the SHS products shown in Figure 7: region 1 (**a**) and region 2 (**b**).

The structures of the SHS products obtained from the mechanically activated Al-Ti-B powder mixture are shown in Figure 7. A qualitative elemental analysis of the structural regions of SHS products is shown in Figure 8.

Based on the XRD results and elemental analysis of local regions of the structure of the SHS products, we found that the SHS products obtained from the Al-Ti-B powder mixture after 60 s of mechanical activation (Figure 7a,b) were structured in the form of separate rectangular and irregularly shaped particles of titanium diboride (region 1), distributed throughout the aluminum matrix (region 2). The particle size in the SHS products varied from 0.02 to 2.5 µm, and the average size was 0.77 µm (Figure 7c). An increase in the mechanical activation duration to 180 s led to a change in the shape of the titanium diboride particles (Figure 7d,e). Mostly isolated particles of irregular or rounded shape were observed. In addition, small aggregates were observed in the structure of the SHS products, formed by recrystallization of titanium diboride particles during the synthesis. The particle size of TiB_2_ ranged from 0.05 to 4 µm, and their average size was 1.5 µm.

The results presented in [26], as well as the results obtained from the present study, allow us to conclude that the change in the shape and size of titanium diboride particles in the SHS products, obtained from a powder mixture of Al-Ti-B with an aluminum concentration of 70 wt.%, along with the change in their distribution throughout the aluminum matrix, is associated with an increase in the reaction surface of particles of the initial components in the Al-Ti-B mixture after increasing the mechanical activation time from 60 to 180 s. The mechanical activation of the mixture for 60 s led to the formation of complex particles containing titanium, boron, and aluminum. During the process of synthesis, aluminum particles heat and form a melt, which prevents the process of recrystallization of titanium diboride particles, and which then leads to their freer growth and the formation of isolated prolate particles uniformly distributed throughout the aluminum matrix. With an increase in the duration of mechanical activation to 180 s, the particles of titanium, boron, and aluminum are crushed more powerfully relative to the particles in the mixture after a mechanical activation of 60 s. This leads to an increase in the reaction surface and in the synthesis temperature. Conversely, a decrease in the size of aluminum particles and an increase in the reaction surface causes the particles of titanium and boron to interact more closely with each other during the synthesis reaction. A denser interaction leads to the recrystallization of titanium diboride and the formation of irregularly shaped particles, the size of which is increased relative to the that of the particles in samples obtained from a powder mixture after 60 s of mechanical activation. An increase in the mechanical activation duration to 900 s also led to a change in the structure of the SHS products. Titanium diboride particles were represented by rounded and irregularly shaped aggregates, which formed an island (skeletal) structure. A similar structure was observed in [15,27]. In addition, the matrix contained isolated irregularly shaped particles distributed between aggregates. The size of particles and aggregates varied in the range of 0.7 to 8 µm, and the average size was 4.3 µm. The change in the structure of the SHS products after 900 s of mechanical activation is associated with the presence of large complex particles in the initial mixture [26]. During synthesis, recrystallization and sintering of titanium diboride particles occurred. Aggregates were formed with an island (skeletal) structure. In addition, pores were found in the structure of all SHS products (Figure 7, regions 3). The formation of pores in the synthesis products can be explained by the presence of impurities (B_2_O_3_, alcohol, O_2_, Cl_2_, N_2_, etc.) in the initial Al-Ti-B mixture, which are released from the sample during combustion.

## 4. Conclusions

In this study, we demonstrated the possibility of obtaining Al-TiB_2_ composite materials using self-propagating high-temperature synthesis and preliminary mechanical activation of the initial Al-(Ti + 2B) mixture with a high aluminum content (70 wt.%). The duration of mechanical activation affects the nature of the reaction front propagation and the synthesis temperature. With a mechanical activation duration of 60 s, a metal matrix structure was formed in the SHS products, consisting of isolated particles of titanium diboride, which were distributed throughout the aluminum metal matrix. An increase in the mechanical activation duration to 180 s led to a change in the shape of titanium diboride particles, from a prolate to an irregular or rounded shape. An increase in the mechanical activation duration to 900 s led to a change in the structure of the SHS products. An island (skeletal) structure was formed, consisting of an aluminum matrix, in which interconnected aggregates and isolated particles of titanium diboride were distributed. Our findings demonstrate that the change in the shape and size of titanium diboride particles in SHS products, obtained from a mixture of Al-Ti-B powders with an aluminum concentration of 70 wt.%, is associated with an increase in the reaction surface of the particles of the initial components in the Al-Ti-B mixture after an increase in the mechanical activation time.

## Figures and Tables

**Table 1:** XRD results of the SHS products.

Mechanical Activation Duration, s	Phases	Phase Content, wt.%	Lattice Parameters, Ǻ	CSR, nm
60	TiB_2_	31	a = 3.0274 c = 3.2261	74
	Al	69	a = 4.0455	66
180	TiB_2_	33	a = 3.0290 c = 3.2285	88
	Al	67	a = 4.0487	95
900	TiB_2_	36	a =3.0256 c = 3.2264	67
	Al	64	a = 4.0447	63
